# Case Report: Efficacy of Pyrotinib in ERBB2 Amplification Pulmonary Adenoid Cystic Carcinoma

**DOI:** 10.3389/fonc.2021.605658

**Published:** 2021-03-18

**Authors:** Zhongben Tang, Feng Lin, Jiarong Xiao, Xiaojun Du, Jian Zhang, Sini Li, Gongshun Tang, Chen Chen, Jian Li

**Affiliations:** ^1^Department of Thoracic, The Affiliated Hospital of Guizhou Medical University, Guiyang, China; ^2^Department of Thoracic, West China Hospital of Sichuan University, Chengdu, China; ^3^Department of Oncology, National Cancer Center/National Clinical Research Center for Cancer/Cancer Hospital, Chinese Academy of Medical Sciences Peking Union Medical College, Beijing, China; ^4^Department of Nuclear Medicine, West China Hospital of Sichuan University, Chengdu, China

**Keywords:** pulmonary adenoid cystic carcinoma, targeted therapy, ErbB2, pyrotinib, stable disease, efficacy

## Abstract

Primary pulmonary adenoid cystic carcinomas are salivary tumors that are low-grade malignant and prone to recurrence and metastasis. Surgery is currently the main treatment, but there is no standard with regard to postoperative adjuvant therapy. Adenoid cystic carcinoma is more sensitive to radiotherapy and patients benefit less from chemotherapy, but few studies have focused on targeted therapy, and their conclusions are inconsistent. With respect to primary pulmonary adenoid cystic carcinoma, large-scale studies cannot be conducted due to its low incidence, and studies on the targeted therapy of it are very scarce. A few case reports indicate that targeted therapy can be effective however, suggesting that it may be a good option. The current report is the first on the occurrence of human epidermal growth factor receptor 2 amplification in pulmonary adenoid cystic carcinoma. The patient was treated with pyrotinib for 6 months and achieved stable disease.

## Introduction

Adenoid cystic carcinoma (ACC) is a salivary tumor. Primary pulmonary ACC (PPACC) is exceptionally rare, and mainly occurs in the trachea or main bronchus. Although it is a low-grade malignant tumor, PPACC is prone to recurrence and metastasis. Surgery is the main treatment at present, but there is no standard with regard to postoperative adjuvant therapy. To date there are very few reports on the targeted therapy of PPACC, but they have important reference value with respect to PPACC treatment. Herein we describe the effects of targeted therapy in a PPACC patient with human epidermal growth factor receptor 2 (ERBB2) amplification, and summarize the current research status of PPACC-targeted therapy.

## Case Description

The patient was a 37-year-old woman who presented with fever. Chest computed tomography (CT) depicted a right middle lung central space-occupying lesion with obstructive pneumonia, and the tumor involved the right lower bronchus. She had no history of smoking, no family history of cancer, no high-risk occupational exposure, and no obvious abnormalities in physical and other examinations. In 2007 she underwent right middle and lower lung resection and lymph node dissection. Pathology results indicated no tumor involvement in the bronchial stump and no lymph node metastasis. The postoperative diagnosis was stage pT2aN0M0 IIa ACC of the right middle lung.

**Figure 1:**
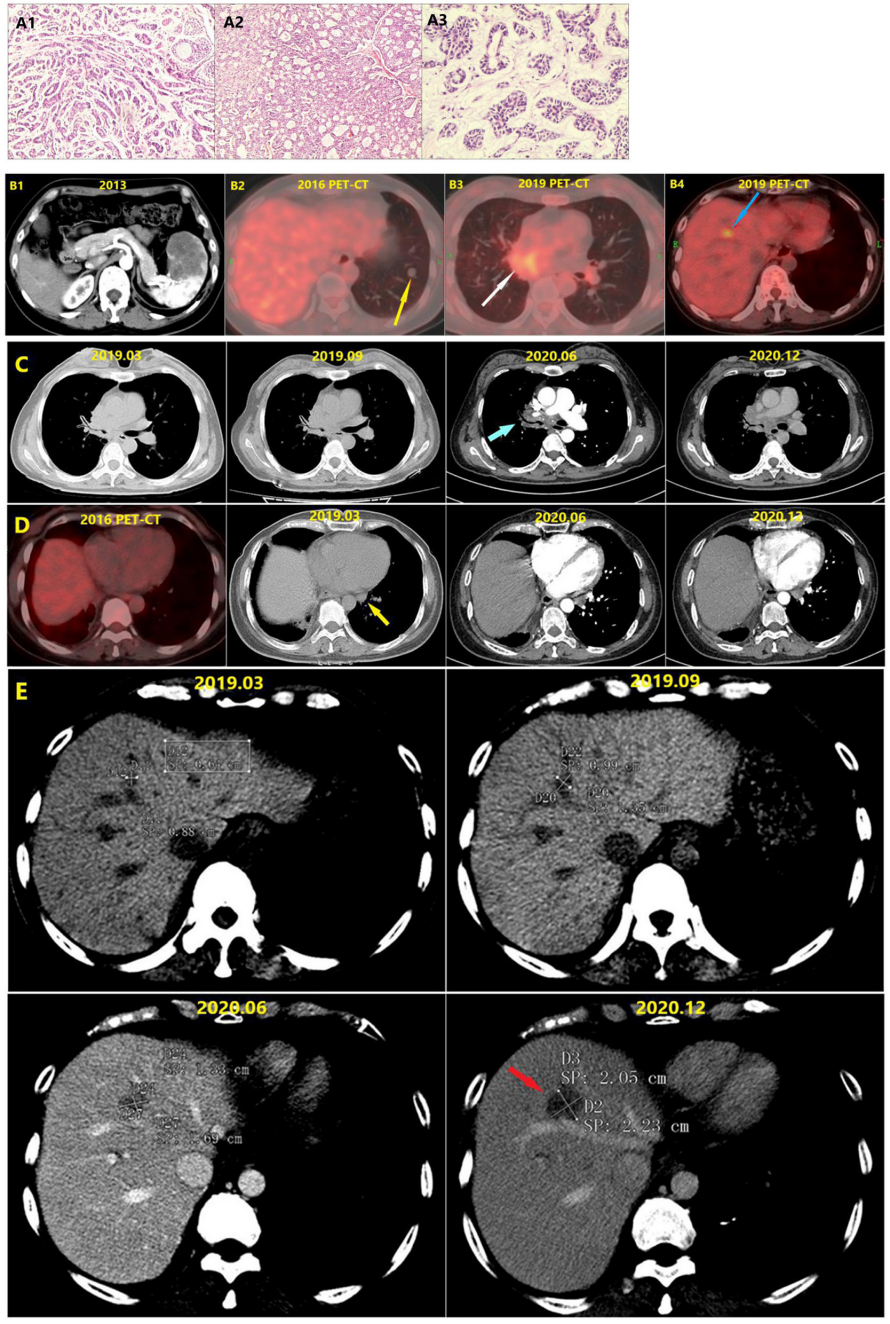
**(A1)** Pathological section of adenoid cystic carcinoma of the right middle lung. **(A2)** Pathological section of the spleen metastasis. **(A3)** Pathological section of the left lower lung metastasis. **(B1)** The spleen metastasis. **(B2)** The left lower lung metastasis (Yellow arrow). **(B3)** The PET-CT in December 2019 showed tumor recurrence in the right hilus pulmonis (White arrow). The bronchoscope finds adenoid cystic carcinoma cells at the end of the right main bronchus. **(B4)** The liver metastasis (Blue arrow). **(C)** Changes of the lesions at hilum of right lung (Blue arrow) in different periods. Comparison of the conditions 9 months before and 6 months after the targeted therapy showed that no significant change occurred to the lesions. **(D)** Changes of the left hilar lymph node in different periods. In 2016, PET-CT showed no lymph node at the hilum of left lung. Comparison of the conditions 9 months before and 6 months after the targeted therapy showed that no significant change occurred to the size of left hilar lymph node (Yellow arrow). **(E)** Changes of liver metastasis (Red arrow) in different periods.

The patient declined postoperative chemotherapy and opted for regular periodic examinations. In 2013 she underwent a splenectomy due to splenic metastasis detected during one of these examinations. In 2016 she underwent wedge resection for left lower lung metastasis due to metastasis of ACC of the left lower lung that was detected via periodic examination (Figures 1A1–A3,B1,B2). In December 2019 CT depicted hepatic nodules, and positron emission tomography-CT suggested recurrence of the right hilar tumor, left hilar lymph node metastasis, and hepatic metastasis (Figures 1B3,B4). At that time the patient underwent bronchoscopy, and ACC cells were detected in the brushing of the end of the right main bronchus, pathologically confirming recurrence. Immunohistochemical results included cytokeratin (+), epithelial membrane antigen (+, part), cytokeratin5/6 (+), P63 (+, part), P40 (+, part), thyroid transcription factor-1 (–), NapsinA (–), CD56 (–), chromogranin A (–), synaptophysin (–), Ki-67 (1%+).

During this period, gene detection was performed via next-generation sequencing using peripheral blood and paraffin sections of tissues from the left lower pulmonary metastasis using the HapOnco 605 panel (approximately 1.31 Mb in total, covering 464 genes) at another hospital. The test used target area capture combined with high-throughput sequencing technology, and 605 tumor-related genes derived from complete or partial exons were analyzed including point mutations, small insertion loss, amplification fusion, single nucleotide polymorphism, and viruses. Detection sensitivity and specificity were above 95%. ERBB2 and cyclin-dependent kinase 4 (CDK4) amplification were evident, as were a number of tumor cells with positive expression of programmed cell death ligand 1 < 1%. In the tissue samples the tumor cells accounted for 90% and the total amount of DNA extracted was 385 ng. In tissues the ERBB2 copy number was 3.2 and the CDK4 copy number was also 3.2. In peripheral blood the ERBB2 copy number was 2.1 and the CDK4 copy number was 2.0.

According to Food and Drug Administration (FDA), China National Medical Products Administration (NMPA) drug specifications, National Comprehensive Cancer Network (NCCN) guidelines, and a comprehensive analysis of the relevant literature the genetic test report recommends trastuzumab (sensitive, evidence grade 3A), T-DM1 (sensitive, evidence grade 3A), and afatinib (sensitive, evidence grade 3A) for ERBB2-amplified non-small-cell lung carcinoma (NSCLC). Piperacillin was recommended for CDK4-amplified NSCLC (sensitive, evidence grade 3B). Due to a lack of relevant studies on targeted therapy for PPACC, and considering the patient's economic situation, we referred to relevant literature on NSCLC and ACC and suggested pyrotinib—a drug made in China used to treat breast cancer and NSCLC with ERBB2 mutation (1–4).

The patient began to take pyrotinib orally (160 mg/tablet, 2 tablets per day) in January 2020. During the treatment period she stopped taking pyrotinib for 20 days due to the coronavirus disease 2019 epidemic, and had no adverse reactions during the treatment. By June 2020, she did not continue the targeted therapy but instead opted for traditional Chinese medicine for personal reasons. She was reexamined 6 months after targeted therapy, and although there was no significant enlargement of the chest lesions during the 21 months before and after targeted therapy, the liver metastatic tumor grew fast. Notably though, the growth rate of the liver metastatic tumor was substantially reduced during the targeted therapy. The total maximum diameters of liver metastase increased by 60% then 43% in two periods 6 months apart before the targeted therapy and after the cessation of targeted therapy. During the 9-month period from September 2019 to June 2021 however (the first 3 months under no treatment and the next 6 months under treatment with pyrotinib), the total maximum diameter of the hepatic metastatic tumor only increased by 25%, indicating that targeted therapy greatly reduced the tumor growth rate (Table 1). Extrapolative calculations suggest that the total growth rate of the maximum diameter was less than 20% during the 6-month period of targeted therapy. According to the Response Evaluation Criteria In Solid Tumors, stable disease was achieved.

**Table 1:** Size changes of liver metastasis at different time.

**Time**	**Lesion size (Sum of diameters) (Unit: cm)**	**The growth rate (time interval)**
2019.03	0.9+0.6 (1.5)	
2019.09	1.4+1.0 (2.4)	60% (6 months)
2020.06	1.7+1.3 (3.0)	25% (9 months)
2020.12	2.2+2.1 (4.3)	43% (6 months)

*(1) During the 6-month Period from March 2019 to September 2019, the patient did not receive any treatment. (2) The Period between September 2019 and June 2020 was 9 months. During this period, the first 3 months under no treatment and the next 6 months under treatment with pyrotinib, the lesion only increased by 25% in the 9-month period however. According to the extrapolative calculations and Response Evaluation Criteria in Solid Tumors (RECIST), the total growth rate of the maximum diameter was less than 20% during the 6-month period of targeted therapy, stable disease was achieved. (3) During the 6-month Period from June 2020 to December 2020, the patient discontinued the targeted therapy, and tried Chinese herbal medicine treatment, the tumor still grew rapidly however (The measurement results are shown in Figures 1C–E)*.

## Discussion

ACC is a type of salivary tumor that usually occurs in the salivary gland in the head and neck. PPACC is rare however, only accounting for ~0.09% of patients with lung cancer (5). It is a low-grade malignant tumor that often develops in the trachea or main bronchus (6). Surgery is the main treatment option for early PPACC, but it has high rates of postoperative recurrence and metastasis (6–9). There is no unified standard for postoperative adjuvant therapy or the treatment of advanced PPACC. Some studies have shown that ACC is sensitive to radiotherapy and that patients benefit less from chemotherapy (10–13).

With the development of targeted therapy, v-kit Hardy-Zuckerman 4 feline sarcoma viral oncogene homolog, epidermal growth factor receptor (EGFR), v-raf murine sarcoma viral oncogene homolog B, Harvey rat sarcoma viral oncogene homolog, Kirsten rat sarcoma viral oncogene homolog, neuroblastoma RAS viral oncogene homolog, phosphatidylinositol-4,5-bisphosphate 3-kinase catalytic subunit alpha, platelet-derived growth factor receptor α, and phosphatase and tensin homolog deleted on chromosome ten gene alterations have been found in ACC (14). In general however, the frequency of gene alternations in ACC is relatively low (15). To date, few studies have focused on targeted ACC therapy, and the conclusions of those studies are inconsistent (16–20).

Because PPACC is rare, reports on gene alterations in PPACC are correspondingly rare. Consequently, there are currently only a few case reports on the targeted therapy of PPACC. Notably though, these reports all suggest that targeted therapy is effective. Song et al. (21) treated a PPACC patient with EGFR exon 19 p.E746-A750 del with icotinib, and the patient maintained progression-free survival for > 19 months. Mendes et al. (22) reported the case of a PPACC patient who was administered erlotinib for the treatment of EGFR exon 21 c.2593G>A, and that patient maintained stable disease for 8 months. Bhattacharyya et al. (23) treated a c-Kit-positive PPACC patient with imatinib and followed the patient up for 1 year, and partial remission was achieved. Fujita et al. (24) treated a patient with advanced PPACC in whom EGFR exon 19 p.L747-T751 del was detected in the exfoliated cells of pleural effusion. The pleural effusion disappeared after the patient was treated with gefinitib, although it was unclear whether gene alterations were from ACC cells. Liu et al. (25) treated a PPACC patient with tamoxifen based on literature reports of patients with ACCs at other locations, and she maintained stable disease for 9 months. Unfortunately that patient did not undergo detection testing for estrogen receptor. Because the low incidence of PPACC precludes large-scale studies, these above-described case reports are a useful resource for putatively evaluating the efficacy of targeted therapy for PPACC.

Herein the occurrence of ERBB2 amplification in a PPACC patient was reported for the first time. During treatment with pyrotinib, the growth of liver metastasis reduced substantially, and stable disease was maintained. Unfortunately, the patient did not continue the targeted therapy but instead opted for traditional Chinese medicine; thus it was not possible to further evaluate the long-term efficacy of the targeted therapy. This is a limitation of this report, but we are continuing to follow the patient up. It was interesting that there was no significant enlargement of the chest lesions during the 15 months before and after targeted therapy, indicating that the tumor grew very slowly in the bronchi. According to the patient's self-reported medical history however, the spleen metastasis and liver metastasis grew very fast. Therefore, we speculated that the growth rate of ACC cells may also be related to the internal environment of the metastasis site, but there are no relevant studies to support this at present.

In conclusion, the current PPACC patient with ERBB2 amplification was treated with pyrotinib and achieved stable disease, indicating that the treatment was effective. In the absence of postoperative adjuvant therapy standards, the observations accompanying treatment in the present patient combined with other relevant reports suggest that targeted therapy can be an effective treatment in PPACC patients.

## Data Availability Statement

The original contributions presented in the study are included in the article/supplementary materials, further inquiries can be directed to the corresponding author/s.

## Ethics Statement

Written informed consent was obtained from the individual(s) for the publication of any potentially identifiable images or data included in this article.

## Author Contributions

ZT and JL drafted the manuscript. FL, JX, XD, JZ, and CC collected the data. ZT, JL, GT, and SL performed the analysis. ZT wrote the manuscript. All authors contributed to the article and approved the submitted version.

## Conflict of Interest

The authors declare that the research was conducted in the absence of any commercial or financial relationships that could be construed as a potential conflict of interest.
